# Microbiome in Female Reproductive Health: Implications for Fertility and Assisted Reproductive Technologies

**DOI:** 10.1093/gpbjnl/qzad005

**Published:** 2023-12-13

**Authors:** Liwen Xiao, Zhenqiang Zuo, Fangqing Zhao

**Affiliations:** CAS Key Laboratory of Systems Biology, Hangzhou Institute for Advanced Study, University of Chinese Academy of Sciences, Hangzhou 310024, China; Beijing Institutes of Life Science/Institute of Zoology, Chinese Academy of Sciences, Beijing 100101, China; Beijing Institutes of Life Science/Institute of Zoology, Chinese Academy of Sciences, Beijing 100101, China; CAS Key Laboratory of Systems Biology, Hangzhou Institute for Advanced Study, University of Chinese Academy of Sciences, Hangzhou 310024, China; Beijing Institutes of Life Science/Institute of Zoology, Chinese Academy of Sciences, Beijing 100101, China; University of Chinese Academy of Sciences, Beijing 100049, China

**Keywords:** Microbiome, Pregnancy, Female reproductive health, Assisted reproductive technology, Microbial biomarker

## Abstract

The microbiome plays a critical role in the process of conception and the outcomes of pregnancy. Disruptions in microbiome homeostasis in women of reproductive age can lead to various pregnancy complications, which significantly impact maternal and fetal health. Recent studies have associated the microbiome in the female reproductive tract (FRT) with assisted reproductive technology (ART) outcomes, and restoring microbiome balance has been shown to improve fertility in infertile couples. This review provides an overview of the role of the microbiome in female reproductive health, including its implications for pregnancy outcomes and ARTs. Additionally, recent advances in the use of microbial biomarkers as indicators of pregnancy disorders are summarized. A comprehensive understanding of the characteristics of the microbiome before and during pregnancy and its impact on reproductive health will greatly promote maternal and fetal health. Such knowledge can also contribute to the development of ARTs and microbiome-based interventions.

## Introduction

With rapid social and economic development, female reproductive health has received increasing attention. There have been various proactive lifestyle recommendations to promote reproductive health, such as engaging in regular exercise and maintaining a balanced diet that includes high-fiber food [1,2]. Recent studies have indicated that the microbiome also plays a crucial role in successful pregnancy and favorable pregnancy outcomes [3,4]. Microbiome refers to the community of all commensal, symbiotic, and pathogenic microorganisms within a body or other environment, and their habitat [5]. Previous studies have shown that the microbiome plays an essential role in the healthy development of the human body and the onset of various diseases [6–8]. Microbiomes in different parts of the human body exhibit different dynamic patterns throughout different life stages, with pregnancy being one of the most critical life stages [9–12]. Previous studies focusing on mothers and babies during the perinatal period have shown frequent microbial interaction and transmission across various body sites, indicating the importance of microbial homeostasis, particularly in the gut and reproductive tract, for the health of both mothers and infants during this critical time window [9,13]. Emerging studies also suggest that the depletion of certain bacteria in early life may lead to significant health issues in childhood [14–16]. Therefore, investigating the characteristics of the microbiome before and during pregnancy, as well as understanding how the microbial variations impact pregnancy health and delivery outcomes, not only contributes to the development of risk prediction models, but also provides targets for microbiome-based interventions to improve reproductive health.

In modern times, unhealthy lifestyles have remarkably increased the incidence of pregnancy complications. These illnesses during pregnancy have a noticeable impact on healthy reproduction. Over the past few decades, the prevalence of infertility has led to the growth of assisted reproductive technologies (ARTs) [17], which provide hope to many couples facing reproductive difficulties. ART refers to any fertility-related treatment in which eggs or embryos are manipulated, with *in vitro* fertilization (IVF) being the main type [18]. The IVF process typically involves retrieving eggs and sperm, fertilizing the eggs, culturing and screening the embryos, and finally transferring an appropriate embryo into the uterus [18]. Recently, a lot of studies have associated the microbiome in the female reproductive tract (FRT) with ART outcomes and provided some interesting observations [19–23]. However, due to inconsistent conclusions among different studies, the progress of ARTs remains limited. A comprehensive understanding of the role of the microbiome in the ART process is crucial and urgently needed.

Pregnancy is a complex process during which several critical changes occur in a woman’s body, including alterations in immune response, hormonal balance, microbial pattern, and metabolism, to support or adapt to the growth and development of the fetus in the uterus [9,11,24]. If these dynamic patterns during pregnancy are disrupted, it can lead to a variety of pregnancy complications such as gestational diabetes mellitus (GDM), preeclampsia (PE), and adverse pregnancy outcomes including premature labor, low birth weight, and macrosomia [25–28]. For example, many studies have confirmed a close association between unfavorable maternal health and the perturbation of metabolites originating from disrupted microbiota during pregnancy [29]. Other observations have linked increased microbial diversity in the reproductive tract to preterm birth, highlighting the potential of the microbiome as an indicator of reproductive health [30,31]. These disorders not only have a significant impact on maternal health, but also have a detrimental effect on the fetus and future infant [32]. Using specific microbes as markers can predict the onset of diseases to a large extent and allow for early intervention [33–35]. At the same time, microbiome-targeted therapies [36], such as probiotic intervention and vaginal microbiota transplantation (VMT), show promise for improving reproductive health and pregnancy outcomes [33,37–39]. Probiotics can enhance the microbial environment and may relieve complications, while VMT aims to restore a normal vaginal microbiome using donor samples, much like fecal microbiota transplantation (FMT) for the gut [39]. These interventions highlight the pressing need to better understand microbial balance in the FRT.

It is of great significance to restore the maternal gut and reproductive tract microbiomes through appropriate interventions to improve maternal health and pregnancy outcomes and promote the healthy development of newborns after delivery [39–42]. More importantly, it is crucial to understand the changes in the microbiome before and after conception and their potential associations and influences on maternal and fetal health. This review provides an overview of the impact of gut and reproductive tract microbiota on female reproductive health before and during pregnancy, and summarizes recent advancements in the microbial characteristics in the FRT. In particular, we emphasize the significance of the microbiome in the IVF process and explore its predictive role in pregnancy complications.

## Impact of lifestyle on gut microbiome and reproductive health

The microbiome is believed to be involved throughout gestation and exerts persistent influences on both mothers and fetuses [9]. Although most host–microbe interactions between mother and offspring occur during pregnancy and delivery, the prepregnancy microbiome of females may also play a vital role in fertility and gestation maintenance [3,4,43]. A number of studies have indicated that maternal conditions before pregnancy persist into the pregnancy and have a tremendous effect on conception and the health of future children [44–46]. Factors such as stress, drug use, smoking, and alcohol consumption, which are known to affect human health, also affect the microbiome of different body sites [46–48].

The intestine is a delicate ecosystem housing trillions of microbes [7]. These microbes interact with each other and play an important role in maintaining gut homeostasis and normal neurodevelopment [6,49,50]. Dysbiosis of the gut microbiota before and during pregnancy is closely associated with some pregnancy complications and pediatric diseases, such as GDM, asthma, allergies, wheezing, and obesity [51–55]. Among these, obesity is one of the most common metabolic disorders that can disturb the gut microbiome and lead to long-term impacts on both mothers and their offspring [56,57]. Obesity induces excessive accumulation of adipose tissue, which leads to chronic inflammation and disrupts metabolic homeostasis [58,59]. It is widely associated with GDM and other diseases [60]. Some studies have demonstrated that mothers with excessive weight gain have a higher chance of giving birth to obese offspring [55,61]. Other studies have also showed that the prepregnancy body mass index (BMI) of mothers is correlated with the healthy condition of their infants, suggesting that the relationship between gut microbiome and reproductive health may begin before conception [62,63]. Nevertheless, the underlying mechanism between the prepregnancy microbiome and postnatal health needs further explanation.

**Figure 1:**
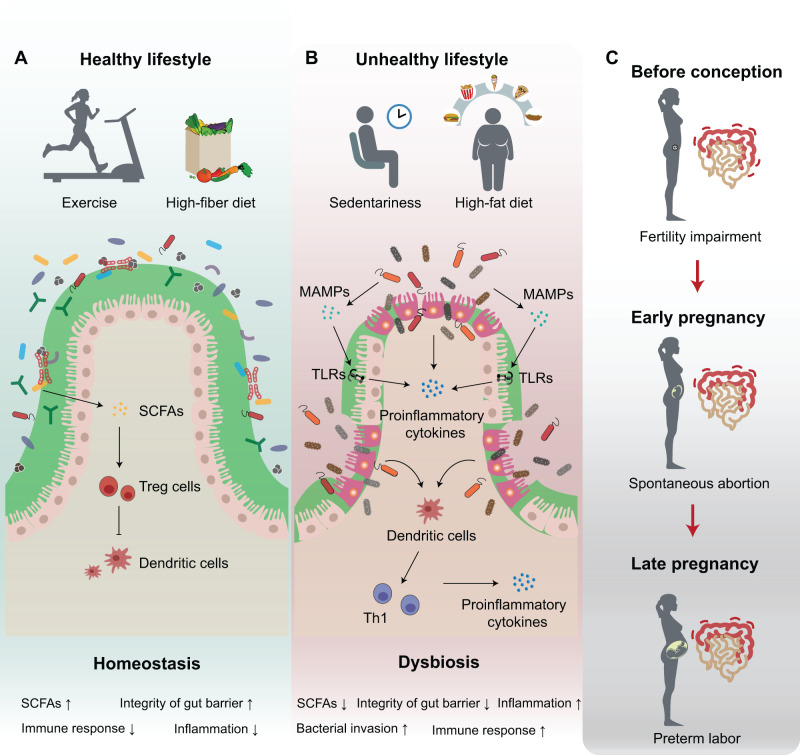
Lifestyle influences gut microbiome and is associated with reproductive health**A**. The impact of healthy lifestyle on gut microbiome and immunity. Active lifestyles such as regular exercise and a high-fiber diet can balance the gut microbiome homeostasis, maintain the gut barrier integrity, and increase the production of SCFAs, which reduce the production of proinflammatory cytokines and down-regulate immune responses and thereby help maintain reproductive health. **B**. The impact of unhealthy lifestyle on the gut microbiome and immunity. Unhealthy lifestyles such as sedentariness and a high-fat diet disrupt the gut microbiome, which further impairs the gut barrier integrity. The evasion of pathogens stimulates immune cells to produce more proinflammatory cytokines, which subsequently activates the immune responses and potentially induces various diseases related to reproductive health. **C**. Persistent inflammation in the intestine at different gestational stages may induce adverse pregnancy outcomes such as fertility impairment, spontaneous abortion, and preterm labor. MAMP, microbe-associated molecular pattern; SCFA, short-chain fatty acid; Th1, T helper type 1; Treg, regulatory T; TLR, Toll-like receptor.

An active lifestyle is recommended for all women, whether currently pregnant or planning for conception [1,64,65]. Numerous studies have illustrated that regular exercise and a balanced diet are powerful contributors to overall health [1,64,66]. The abundance of *Akkermansia* and some butyrate-producing bacterial taxa, such as *Faecalibacterium prausnitzii*, *Roseburia hominis*, and Lachnospiraceae, is increased in women with active lifestyles [67–69]. Such bacteria can produce short-chain fatty acids (SCFAs), which are a common type of bacterial metabolites including acetate, propionate, and butyrate. SCFAs provide more energy to the host and reduce the inflammatory responses in the colonic epithelium [67–69], thereby helping expectant mothers stay in good health (Figure 1A). In contrast, dysbiosis of the maternal gut microbiota enhances the inflammatory responses, increasing the risk of fetal rejection in early pregnancy [70]. Most SCFAs are produced by bacteria from the fermentation of dietary fiber and resistant starch [71]. High fiber can be found in most plant foods such as fruits, vegetables, and grains. A high-fiber diet is believed to increase SCFA levels and improve human health [66]. A recent study involving 120 pairs of GDM women and matched controls from the first trimester to the third trimester indicated that the healthy status of women suffering from GDM was modified with the intake of a high-fiber diet [25]. Parallel changes in the gut microbiome, including *Escherichia coli*, *Fusobacterium mortiferum*, *Bacteroides massiliensis*, and *Bifidobacterium dentium*, were also observed [25].

SCFAs are also one type of the most important bacterial metabolites related to the health of mothers and their offspring [72,73]. A high concentration of SCFAs in obese mother’s gut affects maternal weight gain and glucose metabolism, and is associated with some metabolic disorders such as hypertension and GDM, which may lead to persistent perturbation of the offspring’s microbiome and metabolome [72,74]. Moreover, a high level of SCFAs in the female vagina is associated with infertility, indicating the importance of metabolic balance in the preparation of pregnancy [75]. SCFAs directly or indirectly influence the production of various T lymphocytes, producing a tolerogenic immune environment in infants [76]. One study found that acetic supplementation increased the production of CD4^+^ T cells and the expression of forkhead box P3 (Foxp3), promoting the formation of regulatory T (Treg) cells in early life [77] (Figure 1A). Other studies have also confirmed that SCFAs regulate the function of dendritic cells and Treg cells, reducing inflammatory responses and the development of asthma in offspring [78–81]. The microbiome and its metabolites are widely involved in human health. Apart from SCFAs, other bacterial metabolites such as bile acids (BAs), bioactive lipids or peptides, advanced glycation end products (AGEs), trimethylamine *N*-oxide (TMAO), and imidazole propionate (ImP), have been proven to play essential roles in immune homeostasis. They are closely involved in the regulation of most life activities, exerting either positive or negative influences on mothers and fetuses, the disturbance of which often induces inflammation and specific gastrointestinal disorders [82,83]. Such observations underscore the important role of microbiome homeostasis in women under reproductive age.

Since alteration of gut microbiome is associated with long-term changes in immune response, maintaining gut microbiome homeostasis before and during pregnancy is very important to reproductive health. Consequently, to maintain microbiome homeostasis and stay in good reproductive health, an active lifestyle is necessary for every mother-to-be. For example, bad habits such as being sedentary and a high-fat diet, which can disturb gut microbiome homeostasis and cause an increasing risk of gut inflammation, should be avoided [64]. A high-fat diet is linked to gut microbiota dysbiosis and inflammatory responses [84,85]. Gut microbiome alteration may increase inflammation through microbe-associated molecular patterns (MAMPs) [86]. MAMPs are small molecular motifs conserved within a class of microbes that can be recognized by pattern recognition receptors (PRRs) such as Toll-like receptors (TLRs) in the epithelial cells [86–88]. The activation of TLRs leads to an up-regulation of proinflammatory cytokines [82,87] (Figure 1B). Gut microbiota dysbiosis and inflammation in the colonic epithelium are related to gut leak, which damages the integrity of the intestinal barrier and leads to microbes and bacterial metabolites entering host circulation, inducing infections and diseases [85] (Figure 1B). For example, several studies have indicated that the fertility of women may be impaired when they suffer from inflammatory bowel disease (IBD) [89]. Other studies have also observed a higher risk of pregnancy complications such as spontaneous abortion and preterm labor in women with gut inflammation [90], suggesting an immune interaction between gut and FRT (Figure 1C).

In addition, the microbiota dysbiosis caused by drug abuse before pregnancy also persists for a long time and is very likely to be transmitted to offspring through vertical transmission [53,91]. Recent studies have demonstrated that the inheritance of resistance genes associated with the microbiome, either before or during pregnancy, can lead to antibiotic resistance in offspring and thus increase the risk of asthma and atopic diseases [52,91,92]. However, as there is a great difference in gut microbiome and immune modulation between early pregnancy and late pregnancy, the long-term influences of gut microbiota dysbiosis and the causal relationship between gut microbiome and pregnancy outcomes require further investigation.

## Reproductive tract microbiome of women of reproductive age

The microbiome in the FRT plays an important role in the initiation of conception and exerts influences on pregnancy outcomes, gynecological issues, and fetal development [11,22,31,93,94]. Different from the microbiome in the gut, which is influenced by lifestyle and daily diet, the microbiome in the FRT is more closely related to physiological state, hormone secretion, sexual activities, and hygiene practices [95].

**Figure 2:**
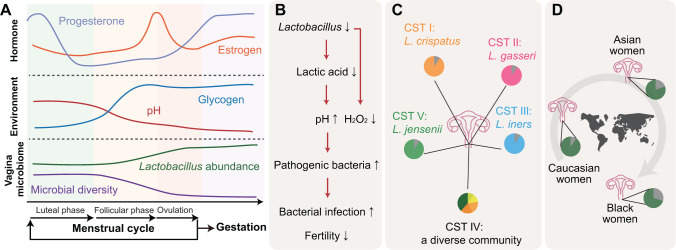
Microbial characteristics in the FRT**A**. Changes in hormones and microbiome before and during pregnancy. As the follicular phase begins, the levels of estrogen and progesterone rise, though the peaks of the two important hormones occur at different stages. Fluctuations in the hormones lead to changes in the vaginal environment and microbiota: an increase in the abundance of *Lactobacillus* spp*.* and a decrease in the vaginal pH and microbial diversity, which gradually makes the vaginal context more favorable for conception. It should be noted that the correct order of a complete menstrual cycle is the follicular phase, ovulation, and the luteal phase. **B**. The role of *Lactobacillus* on fertility. The decrease of *Lactobacillus* is associated with lower lactic acid and H_2_O_2_ levels in the vagina, which further leads to the rise of environmental pH and overgrowth of pathogenic bacteria. The expansion of pathogenic bacteria in the vagina often induces BV and infertility. **C**. Five CSTs in the vagina. Each CST is dominated by different strains. Among them, four CSTs (CST I, CST II, CST III, and CST V) are dominated by *Lactobacillus* spp., while CST IV consists of a diverse microbial community. **D**. Ethnic variations of *Lactobacillus* proportions in the vagina based on limited studies. The vaginal microbiota of most healthy Caucasian women from North America is dominated by *Lactobacillus* spp. [111,117], while Asian and black women appear to have a lower proportion of *Lactobacillus* spp. [111,117]. Nevertheless, further confirmation of this observation is still needed. FRT, female reproductive tract; BV, bacterial vaginosis; CST, community state type.

The vagina is the most widely studied site in the FRT. A healthy female vagina is predominantly colonized by *Lactobacillus* spp. from puberty onwards [31,96]. The normal menstrual cycle in healthy women of reproductive age comprises the follicular phase, ovulation, and the luteal phase, during which the level of hormones and the abundance of microbiome in the vagina fluctuate [38,97]. The cyclical change of hormones and microbiome provides a suitable environment for conception. Estrogen, one of the most important hormones in women’s reproduction, rises during the follicular phase and increases to the highest level at ovulation [95,98] (Figure 2A). Previous studies have indicated that exercise and diet have a significant impact on the levels of estrogen in the body [99–102]. Regular physical activity, especially weight-bearing and resistance exercises, can help increase estrogen levels in females [101,103]. On the other hand, a diet high in processed foods and low in fruits, vegetables, and whole grains can lead to a decrease in estrogen levels [99,100,102]. Maintaining a healthy diet and exercise routine can help regulate estrogen levels and provide numerous health benefits. The rise of estrogen causes glycogen deposits in the vaginal epithelium and thus contributes to the expansion of *Lactobacillus* spp. in the vagina [104] (Figure 2A). In contrast, progesterone does not reach its peak until luteal phase [38]. The high levels of progesterone help thicken the lining of the uterus in preparation for fertilization [105,106]. If there is no fertilized egg, progesterone levels drop and women enter the next menstrual cycle [107].

During pregnancy, the proportion of *Lactobacillus* spp. further increases, reducing the microbial diversity in the vagina (Figure 2A). *Lactobacillus* spp. produce lactic acid [31], the accumulation of which results in a lower pH, creating an unfavorable environment for the proliferation of pathogenic bacteria and other less beneficial bacterial species [95,108]. The abundance of *Lactobacillus* spp. is also associated with the levels of hydrogen peroxide (H_2_O_2_), which has the capability to inhibit the growth of anaerobic bacteria and enhance the competitiveness of *Lactobacillus* spp. [31,95] (Figure 2B). The perturbation of the vagina microbiome may disturb the homeostasis of the vaginal microenvironment. Previous studies have demonstrated that pregnant women with a higher microbial diversity in the vagina have an increased risk of miscarriage and preterm birth [109,110]. In contrast, women with a dominance of *Lactobacillus* spp. experience less infection and lower levels of inflammation [75]. Studies have also implied that infants acquire their initial microbiome from the maternal vagina and babies delivered via the vagina experience fewer diseases such as asthma and allergies [9,13–15], indicating the importance of inheriting a beneficial maternal microbiome.

However, the populations of lactobacilli exhibit a high intraspecies diversity, and not all strains of *Lactobacillus* in the vagina provide equivalent protective effects on fertilization and pregnancy outcomes [31]. According to recent studies based on large populations, there are five microbial clusters, namely community state types (CSTs), in the healthy female vagina [30,111]. CST I, CST II, CST III, and CST V are dominated by species *Lactobacillus crispatus*, *Lactobacillus gasseri*, *Lactobacillus iners*, and *Lactobacillus jensenii*, respectively [30,31,111] (Figure 2C). Among them, CST I with the dominance of *L*. *crispatus* provides the most protective benefits to the host health, while CST III dominated by *L*. *iners* offers the least [31]. The other two communities (CST II and CST V) are moderate and are much closer to the former [31]. The different effects among *Lactobacillus* strains may originate from their metabolic capabilities [31]. For example, among the four bacteria, only *L*. *iners* has no capability to produce D-lactic acid and H_2_O_2_, which are thought to have the ability to inhibit the overgrowth of anaerobic bacteria and are involved in the modulation of the immune system [112–114]. Nevertheless, the deeper reasons behind the problem may need further exploration. Another reason why CST III (*L*. *iners* dominant community) is not favorable in the vaginal microenvironment is that this community is less stable and often transitioned to CST IV [30,31]. CST IV is a community that lacks lactobacilli and harbors a higher ratio of strictly anaerobic genera including *Prevotella*, *Dialister*, *Atopobium*, *Gardnerella*, and *Sneathia* [31] (Figure 2C). Such a community is characterized by a much higher vaginal pH (> 4.5) and is often associated with the occurrence of bacterial vaginosis (BV), which has associations with infertility and spontaneous abortion [31,95].

While multiple studies have confirmed the negative effects of CST IV on reproductive health, the relationship is not absolute, as most published studies have primarily focused on Western populations. Limited literature focusing on black women and Asian populations has revealed that the proportion of *Lactobacillus* spp. in a healthy vagina may vary depending on ethnicity [111,115–121]. For example, cross-population studies conducted by Zhou et al. and Ravel et al. demonstrated that healthy black women from North America harbor the highest proportion of non-*Lactobacillus* species in the vagina, followed by Asian and Caucasian women [111,117] (Figure 2D). Whether this observation on different populations is universal remains unknown. The underlying mechanism of variation of microbial composition in the female vagina across different ethnic groups and its further influences need more evaluation. Nonetheless, the divergence in the vaginal microbiome community in different races indicates that some gynecological disorders are associated with global microbiota dysbiosis rather than the emergence or absence of a single microbe. There is still a long way to go to thoroughly understand the exact relationship between vaginal microbiome and reproductive health.

## Reproductive tract microbiome and ARTs

The decline in fertility in both males and females and the trend of delaying pregnancy to a later age have promoted the development of ARTs [17]. However, the moderate success rate of pregnancy and the unclear mechanisms behind it are hindering the wide use of ARTs [122]. In addition, some studies observe a higher risk of adverse outcomes in ART-conceived babies (and animals) than in naturally conceived ones [123–126], pushing us to update our understanding in this area.

**Figure 3:**
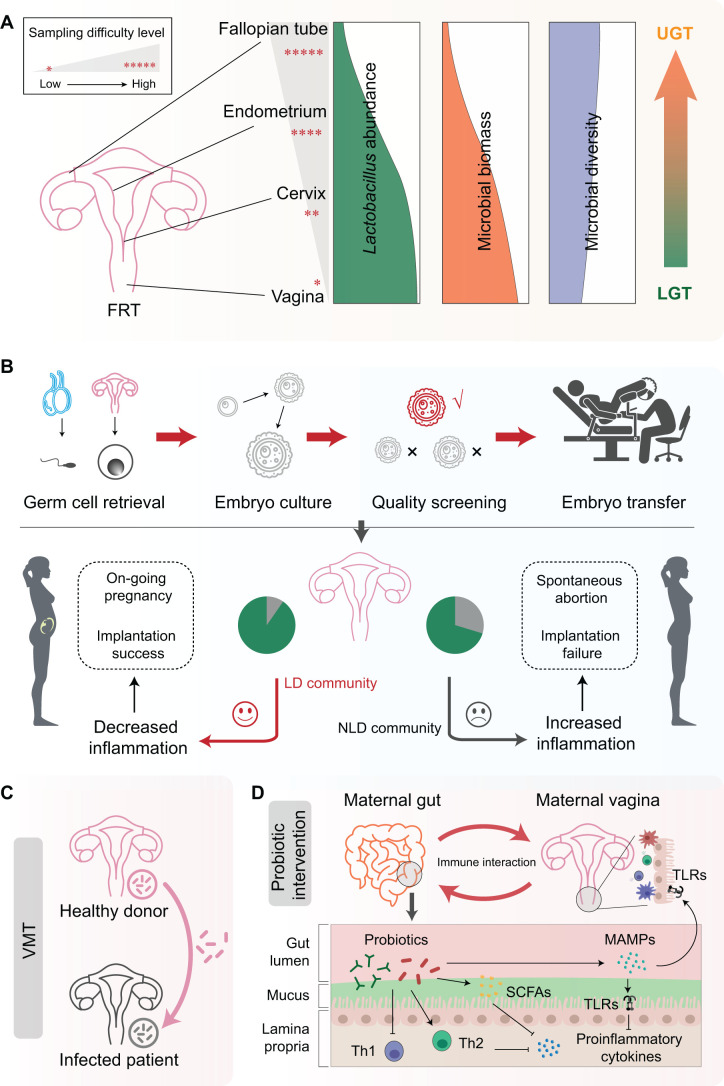
Microbiome in ARTs and interventions**A**. Difference of microbiomes in FRT. The proportion of *Lactobacillus* spp. and microbial biomass sharply decrease from LGT to UGT, while the alpha diversity of microbiota and sampling difficulty increase. **B**. General flow of IVF and the impact of different microbial communities on ART outcomes. The IVF process typically involves retrieving eggs and sperm, fertilizing the eggs, culturing and screening the embryos, and finally transferring an appropriate embryo into the maternal uterus. Different microbial communities are associated with different outcomes. Specifically, an LD community is considered to be related to decreased inflammation and more favorable ART outcomes. **C**. Restoration of vagina microbiome using VMT. VMT is a novel treatment strategy aiming at modifying vaginal microbiota composition and treating vaginal dysbiosis and related conditions. It involves transferring vaginal fluid from a healthy donor to a recipient to reestablish a normal vaginal microbiome. **D**. Probiotic intervention and immune interaction between the gut and vagina. Probiotics, especially lactic acid-producing bacteria, can positively modulate immune responses in the gut and vagina. This includes stimulating Th2 cells and suppressing Th1 cells, altering cytokine profiles, and modulating tolerance to commensal bacteria, which helps create an optimal immune environment in the vagina that supports healthy microbiota. ART, assisted reproductive technology; IVF, *in vitro* fertilization; LD, *Lactobacillus*-dominated; LGT, lower genital tract; NLD, non-*Lactobacillus*-dominated; UGT, upper genital tract; Th2, T helper type 2; VMT, vagina microbiota transplantation.

The role of the microbiome in the FRT and its related modulation of the immune system on the success of pregnancy have been highlighted in recent years, promoting advances in exploring the impact of microbiome on ARTs [19,20,127]. The most abundant bacteria in the healthy FRT are *Lactobacillus* spp.; however, the proportion of *Lactobacillus* spp. varies between the lower genital tract (LGT; vagina and cervix) and the upper genital tract (UGT; uterus, fallopian tubes, and ovaries) [93,98,128]. For example, during a normal pregnancy, there are about 99% of bacteria belonging to *Lactobacillus* in the vagina, but the proportion of such lactic acid-producing bacteria is much lower in the cervix, endometrium, and fallopian tubes [93,128] (Figure 3A). Studies have also indicated that the microbial biomass in the UGT largely decreases, but the microbial diversity slightly increases compared to that in the LGT [20,93,98,128] (Figure 3A). Although different locations of the FRT have distinct microbial compositions, the protective effect of *Lactobacillus* spp. on ART outcomes seems comparable. Previous studies have linked the vaginal microbiome to ART outcomes [129–137]. Vagina microbiota dysbiosis, which is mainly characterized by a shift in microbial community from other CSTs to CST IV, can induce BV [20,31]. In women receiving ART, BV is associated with implantation failure and spontaneous abortion [138]. In contrast, women with a high abundance of *Lactobacillus* spp. in the vagina are more likely to become pregnant during ART [19].

Like in the vagina, the most prevalent colonizer in the cervix is *Lactobacillus* spp. (97%–99%). Apart from this, the cervix also harbors a small proportion of non-*Lactobacillus* bacteria [128], such as *Gardnerella*, *Streptococcus*, *Prevotella*, and *Pseudomonas*, while the total biomass in the cervix is much lower than that in the vagina [139,140] (Figure 3A). Different from the vagina and the cervix, the UGT, such as the uterine cavity and fallopian tubes, contains a much lower biomass of microbes [20,98,141] (Figure 3A). In addition, the more invasive and complicated sampling process increases the risk of contamination, posing great challenges to exploring the role of the microbiome in such locations [38]. Despite its controversy, some studies have examined microbes in the UGT. For example, the endometrial microbiota is thought to consist of 30%–70% *Lactobacillus* and other bacteria such as *Gardnerella*, *Bifidobacterium*, *Flavobacterium*, *Pseudomonas*, *Streptococcus*, *Prevotella*, and *Atopobium*, which vary dependent on the populations [128,134,142,143]. Some studies have indicated that the cervical and endometrial microbiomes might be involved in ART outcomes, influencing the implantation of embryo and fetal development [20,21].

Similar to that in the vagina, *Lactobacillus* spp. provide a protective effect on the cervix and endometrium as shown in most studies [22,23,137,142–152]. Decreased *Lactobacillus* abundance and increased microbial diversity in the cervical microbiota are positively associated with adverse ART outcomes [22,23,137,143–150]. The increase of non-*Lactobacillus* bacteria such as *Gardnerella* may be associated with human papillomavirus (HPV) risk in women, inducing inflammation and cervical cancer [151]. Studies including hundreds of women undergoing ART have also demonstrated that compared with *Lactobacillus*-dominant (LD) microbiota, women with non-*Lactobacillus*-dominant (NLD) communities in endometrium exhibit lower rates of implantation, pregnancy, ongoing pregnancy, and live birth [22,146]. Increased endometrial blooms of non-*Lactobacillus* bacteria, such as *Gardnerella*,* Streptococcus*,* Staphylococcus*, and *Enterobacteriaceae*, are associated with chronic endometritis and endometriosis, which increases the risk of infertility and implantation failure [142,143,152].

Microbes in the fallopian tubes or ovaries exhibit much lower *Lactobacillus* abundance and much higher inter-individual variability, and their relationship to ART outcomes is controversial [20,93] (Figure 3A). For example, some studies claimed that extremely low or even no *Lactobacillus* spp. existed in the fallopian tubes or ovaries [128,153], while other studies detected an LD community and associated the presence of *Propionibacterium* and *Actinomyces* in such locations with adverse reproductive outcomes [154,155]. However, due to the lack of sufficient evidence, further studies should be conducted to investigate the causal relationship between ART outcomes and the microbiome in the fallopian tubes or ovaries.

Pregnancy is a very complicated process, during which women undergo great changes in hormones, microbiome, metabolites, and immune responses [9,20]. Therefore, to promote the success of IVF, many factors need to be considered. The success of conception is believed to be associated with the development of tolerance by the maternal immune system towards the fetus [156]. The beginning of pregnancy is accompanied by great changes in both innate immunity and adaptive immunity [127,157,158]. Changes in immune responses are synchronized with corresponding hormonal fluctuations and microbiome alterations, accepting or rejecting the implantation of the embryo during ART [157] (Figure 3B). For example, during normal pregnancy, the levels of estrogen and progesterone increase, which leads to further reduction of the production of proinflammatory mediators such as nitric oxide (NO), tumor necrosis factor-α (TNF-α), and interferon-γ (IFN-γ) [157]. The increase of progesterone forms an LD community in FRT, which not only increases the production of lactic acid, but also down-regulates the expression of TLR4, suppressing inflammatory responses in cervicovaginal epithelial cells [75,156]. The LD community in FRT at early pregnancy can be a beneficial factor in preventing fetal rejection during ART [20,21]. In contrast, the NLD community is frequently associated with extensive inflammatory responses, leading to embryo transfer failure in many ART cases [20,21]. Despite such importance of microbiome in the FRT, the ART outcomes can be associated with extensive factors beyond microbiota, such as embryo types (frozen or fresh) and quality, maternal age, hormonal level, and standardized approach [95]. Further studies are needed to enhance our current understanding of the underlying immune mechanism behind the associations between the microbiome and reproductive outcomes in ARTs.

Numerous diseases have been associated with the microbiome [35]. Considering the vital role of the FRT microbiome in reproductive health, some studies have tried to restore vaginal microbiota to alleviate the impact of gynecological disorders on reproduction [37,39–42,159–162]. One solution is microbiota transplantation. Microbiota transplantation is a strategy to transfer microbiota from a healthy donor to the patient to change the recipient’s microbial composition and confer a health benefit. FMT has been used to treat many diseases such as obesity, recurrent *Clostridium difficile* infection, and IBD, achieving favorable outcomes [163,164]. Recently, several studies have proposed using VMT, which is similar to FMT, to reconstruct the vaginal microbial community [39,42,162] (Figure 3C). A full long-term remission has been reported in some women with FRT infection, indicating that the restoration of vaginal microenvironment is beneficial to the reproductive health.

Apart from VMT, some studies have suggested the existence of microbiome–gut–vagina axis, leading to the intervention using probiotics to rehabilitate gut microbiota homeostasis and thereby improve reproductive health [37,40,41,159–161]. Strains in most probiotic supplements such as *Lactobacillus* spp. are involved in balancing SCFA levels and regulating the balance of T helper type 1 (Th1) and Th2 cells [165–168] (Figure 3D). The elevated production of SCFAs and decreased Th1/Th2 cell ratio suppress the production of proinflammatory cytokines and enhance the integrity of the uterine barrier, further reducing the risk of many reproductive disorders, including infertility, BV, endometrial diseases, polycystic ovary syndrome (PCOS), GDM, PE, and preterm labor both in IVF and natural conception [41,161,166,169,170]. Probiotic-derived MAMPs also have the ability to modulate the expression of TLRs and alleviate inflammatory responses in epithelial cells [159,160,171,172] (Figure 3D). Intervention with probiotics in females has been proven to restore the microbiome in both the gut and FRT, improving the health of mothers and offspring [41,173]. Nevertheless, there still are some failure cases in probiotic intervention, suggesting the urgent need to evaluate the safety of probiotics for pregnant women and to explore the molecular mechanism underlying microbiome in reproductive health [40,174].

## Microbial biomarkers and pregnant complications

A number of studies have highlighted the importance of microbiome in the health of pregnant women and their unborn children [9,11,55,175,176]. Previous investigations have demonstrated the changes in the microbiome and immune responses at different body sites during normal pregnancy [9,177]. In early pregnancy, commencing from conception to the end of the 13th week of pregnancy, the microbiome and immunity in both the gut and vagina are similar to that in healthy unpregnant women [9,178]. While in the third trimester, which ranges from the 27th week to delivery, the microbial profiles and immune responses resemble those of women suffering from obesity [177]. Studies indicated that the increase of Enterobacteriaceae, *Streptococcus*, and *Lactobacillus*, as well as high levels of insulin insensitivity and blood glucose, helps mothers harvest more energy, thus supporting the growth of fetuses [172,177]. Nevertheless, an unhealthy lifestyle during pregnancy disturbs the maternal microbiome, which is associated with poor maternal conditions and leads to the occurrence of pregnancy complications, further influencing the development of infants [13,25,26,175].

With current knowledge and case-control studies, bacterial biomarkers can be identified to promote the prevention and early diagnosis of many pregnancy complications. Previous studies have associated the microbiomes in the female gut and reproductive tract with some obstetric and gynecological diseases, such as recurrent miscarriage (RM) [179], repeat implantation failure (RIF) [22,134,146,148,180], PCOS [29,181–192], GDM [25,175,193–199], gestational hypertension [200], PE [27,201–209], preterm labor, and preterm premature rupture of membrane (PPROM) [26,210–214]. While some studies reported consistent observations for certain diseases, other studies reached controversial conclusions. For example, GDM is one of the most common metabolic syndromes during the second trimester, which is often associated with dysbiosis of the gut microbiota and metabolic alteration [175,215]. Crusell et al. demonstrated that mothers with GDM exhibited a higher abundance of *Faecalibacterium* and *Anaerotruncus* and a decreased abundance of *Clostridium* and *Veillonella* in the gut [193]. In contrast, Cortez et al. indicated that the gut microbial differences were insignificant between GDM and healthy pregnant women [194]. Differences in ethnicity and environments can be one of the reasons that result in such controversial observations, while further associations remain explored. A prospective study conducted by Sun et al. demonstrated significant differences in the gut microbiome, including *Ruminococcus bromii*, *Alistipes putredinis*, and *Bacteroides ovatus*, between GDM women and their healthy controls [25]. Furthermore, they indicated that the microbial characteristics at early pregnancy can be used to predict the occurrence of GDM in the future [25]. Nevertheless, regarding preterm labor, a more uniform relationship is observed. Most studies have confirmed the protective effect of *L*. *crispatus* in the vagina, while *L*. *iners* is one of the most common taxa associated with low gestational age [26,210–214].

Previous studies have indicated that persistent infections within the vagina or uterine cavity might increase the risk of pregnancy loss and lead to unfavorable outcomes [216,217]. In addition, communication of microbes between the vagina and uterine cavity is associated with reproductive health. A recent study has demonstrated that some bacteria in the vagina, such as *Clostridium perfringens* and *Prevotella bivia*, may ascend to the uterine cavity and induce inflammatory responses [94]. In contrast, other microbes such as *Lactobacillus murinus* can provide a protective effect during microbial translocation between the vagina and the uterus [94]. Such explicit bacterial biomarkers could largely reduce adverse outcomes during pregnancy in clinical settings. Recent studies have also implied that the microbiomes at different body sites might experience disturbance when mothers are under unfavorable conditions, highlighting the close relationship between the microbiome and reproductive health. Wang et al. showed that significant perturbance of microbiomes in the maternal gut, vagina, and oral cavity was observed in GDM women, and the microbial interactions of key bacteria with others were closely associated with maternal health [175]. In addition, concordance of microbial dysbiosis was also found between mothers and their future babies [175], indicating the predictability of microbiome during pregnancy on the health of mothers and infants. Pregnant women with periodontal infections were also found to have a higher risk of hypertension or preterm labor during pregnancy [200,218,219]. Detection of a high proportion of pathogens, such as *Porphyromonas gingivalis*, in both subgingival plaque and genital tract suggested that bacteria may enter maternal circulation and further influence maternal health and fetal development [200,218].

The human body is a balanced ecosystem, and alterations in the microbiome are associated with various changes in metabolites and immune responses [11,50], making it a mirror of human health and predicting future health for both mothers and offspring. However, the use of microbial biomarkers to predict and prevent pregnancy complications has some limitations. Sampling the reproductive tract is considerably more challenging compared with other sites such as the oral cavity and stool, which introduces risks of contamination. In addition, various confounding factors, including hormone changes and diet influences during pregnancy, may also introduce bias into taxonomic profiling. Moreover, it is important to note that most previous studies on microbial biomarkers were observational, establishing associations rather than causation. Consequently, caution must be taken to avoid unintended outcomes when using microbial biomarkers. Confirming the causal relationships between the microbiome and various diseases is still necessary before adopting microbiome-based therapies for pregnancy complications and childhood disorders. To achieve this, the underlying mechanisms of specific probiotic strains and microbial communities should be further explored in animal models. Large clinical cohorts focusing on major diseases are also needed to establish causal relationships in humans. In summary, although microbiome research presents promising possibilities for understanding, diagnosing, and treating disorders of the FRT, cautious interpretation and understanding causal relationships are important to meaningful clinical translation. Robust basic, translational, and clinical research will be essential to fully realize the potential benefits of microbiome-based therapies for maternal and infant health.

## Conclusion and perspectives

This review summarizes the long-term impacts of various lifestyles on the microbiome and reproductive health. It highlights the association between healthy habits and the microbiome before and during pregnancy and emphasizes the important role of the FRT microbiome in predicting pregnancy outcomes. Further understanding of the microbiome during pregnancy will greatly advance biological and medical research, increase the success rate of ARTs, and reduce the risk of adverse pregnancy outcomes.

## CRediT author statement

**Liwen Xiao:** Writing – original draft, Visualization. **Zhenqiang Zuo:** Writing – review & editing, Visualization. **Fangqing Zhao:** Conceptualization, Supervision, Writing – review & editing. All authors have read and approved the final manuscript.

## Competing interests

The authors have declared no competing interests.
